# Multi-omics analysis reveals that ornithine decarboxylase contributes to erlotinib resistance in pancreatic cancer cells

**DOI:** 10.18632/oncotarget.21572

**Published:** 2017-10-06

**Authors:** Won-Jun Jang, Boyeon Choi, Sang-Hoon Song, Naeun Lee, Dong-Joon Kim, Sooyeun Lee, Chul-Ho Jeong

**Affiliations:** ^1^ College of Pharmacy, Keimyung University, Daegu 42601, Republic of Korea; ^2^ China-US (Henan) Hormel Cancer Institute, Zhengzhou 450008, China

**Keywords:** resistance, erlotinib, pancreatic cancer, metabolomics, ornithine decarboxylase

## Abstract

Molecular and metabolic alterations in cancer cells are one of the leading causes of acquired resistance to chemotherapeutics. In this study, we explored an experimental strategy to identify which of these alterations can induce erlotinib resistance in human pancreatic cancer. Using genetically matched erlotinib-sensitive (BxPC-3) and erlotinib-resistant (BxPC-3ER) pancreatic cancer cells, we conducted a multi-omics analysis of metabolomes and transcriptomes in these cells. Untargeted and targeted metabolomic analyses revealed significant changes in metabolic pathways involved in the regulation of polyamines, amino acids, and fatty acids. Further transcriptomic analysis identified that ornithine decarboxylase (ODC) and its major metabolite, putrescine, contribute to the acquisition of erlotinib resistance in BxPC-3ER cells. Notably, either pharmacological or genetic blockage of ODC was able to restore erlotinib sensitivity, and this could be rescued by treatment with exogenous putrescine in erlotinib-resistant BxPC-3ER cells. Moreover, using a panel of cancer cells we demonstrated that ODC expression levels in cancer cells are inversely correlated with sensitivity to chemotherapeutics. Taken together, our findings will begin to uncover mechanisms of acquired drug resistance and ultimately help to identify potential therapeutic markers in cancer.

## INTRODUCTION

Pancreatic cancer is one of the most fatal diseases, with an annual incidence that almost equals its annual mortality rate [1, 2]. The overall five-year survival rate is less than 5% and most patients die within 6 months of diagnosis [3]. This poor outcome is largely due to late diagnosis and the aggressive characteristics of the cancer, including metastasis and resistance to chemotherapeutic drugs [4].

Epidermal growth factor receptor (EGFR) is overexpressed in up to 60% of human pancreatic cancers [5], therefore blocking the EGFR pathway could be a promising strategy for treating pancreatic cancers. In fact, combination therapies using erlotinib, a small molecule tyrosine kinase inhibitor of EGFR (EGFR-TKI), together with gemcitabine, has been clinically approved for the treatment of both unresectable locally advanced and metastatic pancreatic cancer. However, although this regimen has prolonged the median survival time of patients with pancreatic cancer, it has limited therapeutic benefits because 25-30% of patients became drug-resistant after only a short course of therapy [6–8].

Overcoming drug resistance will require a better understanding of the molecular mechanisms that underlie the complex biological pathways involved in acquiring this resistance in pancreatic cancer. The application of metabolomics to cancer research could help to explain the causes of phenotypic changes in cancers during acquisition of resistance to chemotherapeutics. A recent metabolomic study enhanced our understanding of gemcitabine resistance in pancreatic cancer cells [9]. Analyzing overall metabolic and transcriptomic changes in cancer cells has enabled us to identify biological pathways that contribute to acquired drug resistance. In this context, multi-omics analysis, including metabolomics and transcriptomics, is becoming an increasingly popular tool for understanding interrelations between different metabolites, biological molecules, and the dynamics of biological systems in cells [10, 11].

In the present study, we established an erlotinib-resistant pancreatic cancer cell line, BxPC-3ER, from parental BxPC-3 cells, and characterized their resistance-related phenotypes, such as sensitivity to erlotinib, anchorage-independent cell growth, invasiveness, and epithelial-mesenchymal transition (EMT). This multi-omics analysis was also used to identify metabolic and molecular alterations during the acquisition of erlotinib resistance. Specifically, metabolomic profiles of erlotinib-sensitive BxPC-3 and -resistant BxPC-3ER cells were examined by untargeted and targeted metabolomics analyses using mass spectrometry (MS). Moreover, transcriptomic analysis was performed to determine relationships between metabolites and potential enzymes involved in erlotinib resistance in pancreatic cancer cells. Together, these multi-omics based approaches will begin to unravel the molecular mechanisms that underlie acquired resistance to chemotherapeutics, and identify novel targets with which to develop strategies to overcome this resistance.

## RESULTS

### Erlotinib resistance in BxPC-3ER cells is associated with increased anchorage-independent cell growth, invasiveness, and EMT

**Figure 1:**
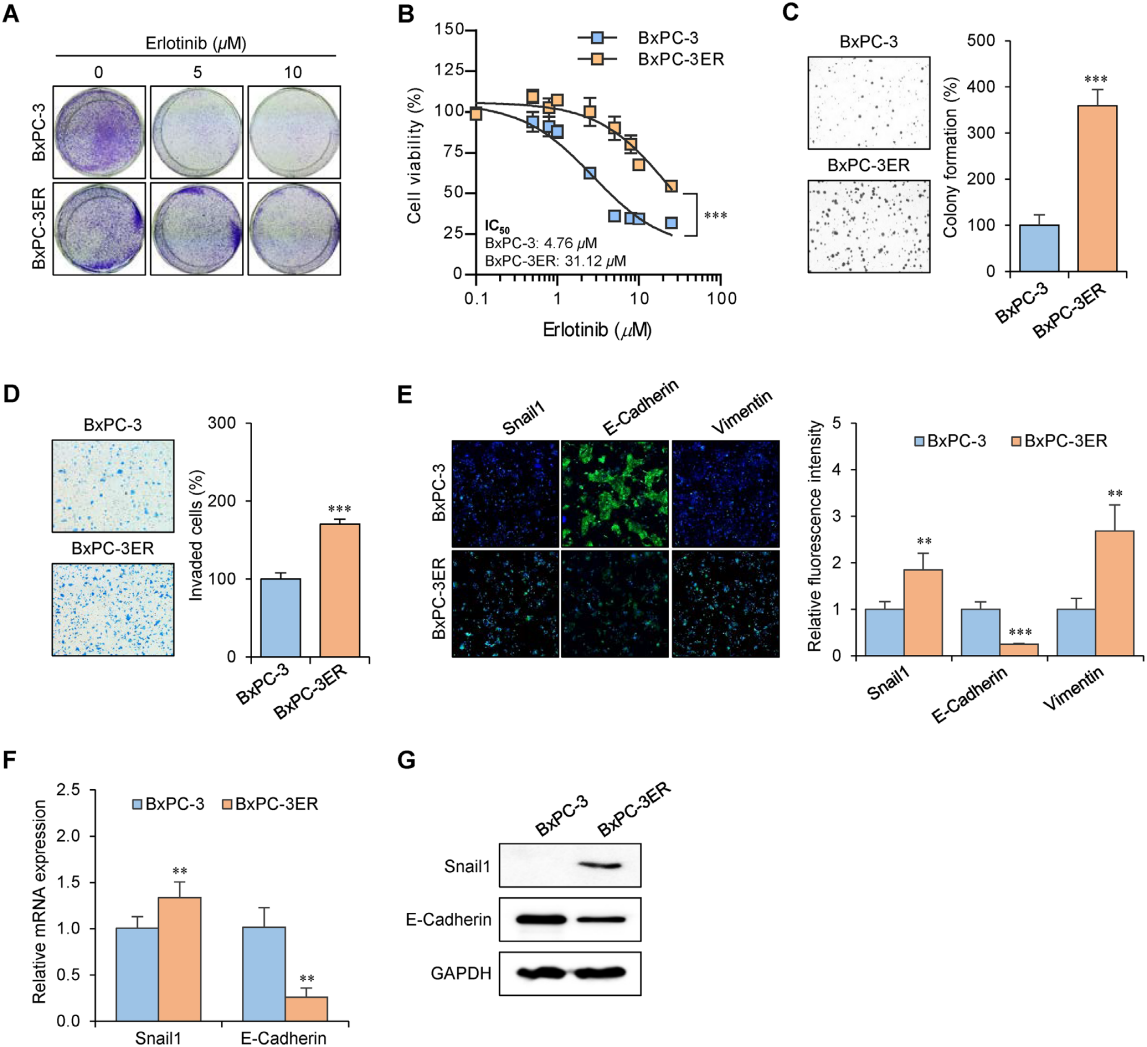
Acquired erlotinib resistance in BxPC-3ER cells is associated with increased invasiveness and EMT **(A)** BxPC-3 and BxPC-3ER cells were plated onto 6-well plates (1.5×10^5^ cells/well) and treated with indicated concentrations of erlotinib for 72 h. Cells were stained with crystal violet and photographed. **(B)** Viability of erlotinib-treated BxPC-3 and BxPC-3ER cells. Cells were seeded onto 96-well plates (1×10^3^ cells/well) and treated with various concentrations of erlotinib for 72 h. Cell viability was determined using an MTT assay. Statistical analysis was conducted using two-way ANOVA. ^***^p < 0.001, compared to BxPC-3 cells. Error bars show mean ± SD (n = 4). IC_50_ was determined by nonlinear regression using GraphPad Prism software. **(C)** Anchorage-independent growth of BxPC-3 and BxPC-3ER cells. Cells were seeded onto 6-well soft agar plates (1×10^4^ cells/well) and incubated for 7 days. Colony images were obtained using a light microscope. Random areas in colonies grown in soft agar were scanned (nine areas per well, three wells per set). Error bars represent mean ± SD (n = 27). Statistical significance was determined using a Student's *t*-test (^***^p < 0.001). **(D)** Invasiveness of BxPC-3 and BxPC-3ER cells. Cells were plated onto Matrigel-coated transwells (24-well). Live cells that invaded the lower surface were fixed, stained and manually counted using a microscope. Statistical analysis was conducted using a Student's *t*-test. ^***^p < 0.001, compared with the BxPC-3 group. Error bars show mean ± SD (n = 4). **(E)** Immunofluorescent staining of EMT markers in BxPC-3 and BxPC-3ER cells. Cells were incubated with indicated antibodies and stained with DAPI. Immunofluorescent cells were observed using a fluorescence microscope and signal intensity was quantified using ImageJ software and normalized to DAPI. Error bars represent mean ± SD (n = 5). Statistical significance was determined using a Student's *t*-test. ^**^p < 0.01; ^***^p < 0.001 versus BxPC-3 cells. **(F)** Quantitative real-time PCR analysis. GAPDH was used for normalization. Error bars show mean ± SD (n = 4). Statistical significance was determined using a Student's *t*-test. ^**^p < 0.01 versus BxPC-3 cells. **(G)** Whole cell lysates were assayed by western blot using antibodies against Snail1 and E-cadherin. GAPDH was used as a loading control.

Erlotinib-resistant BxPC-3 (BxPC-3ER) cells were established from parental BxPC-3 cells via continuous exposure to erlotinib for over 6 months. Microscopic analysis revealed that BxPC-3ER cells exhibited a distinct spindle-shaped morphology, with increased numbers of pseudopodia (Supplementary Figure 1A). Also, the growth rate of BxPC-3ER cells was about 30% slower than that of BxPC-3 cells from 2 days after seeding (Supplementary Figure 1B). Crystal violet staining revealed that erlotinib treatment (5-10 μM) resulted in a dramatic decrease in the number of viable BxPC-3 cells compared to BxPC-3ER cells (Figure 1A). The half maximal (50%) inhibitory concentration (IC_50_) values for erlotinib in BxPC-3 and BxPC-3ER cells were found to be 4.76 μM and 31.12 μM, respectively, which verified the acquisition of resistance to erlotinib in BxPC-3ER cells (Figure 1B). Next, we determined the ability of cells to form colonies in soft agar. Our data revealed that BxPC-3ER cells displayed a more metastatic phenotype, with an increased ability to form colonies in soft agar compared to their parental cells (Figure 1C). We also performed a cell invasion assay using transwell chambers and serum-free media without a chemoattractant. As shown in Figure 1D, BxPC-3ER cells displayed increased invasiveness through Matrigel compared to BxPC-3 cells. These results indicate that erlotinib-resistant BxPC-3ER cells exhibit enhanced anchorage-independent cell growth and invasiveness versus their parental cells, suggesting a close correlation between erlotinib resistance and metastatic potential in BxPC-3ER cells. Based on a previous report [12], we checked whether the acquired erlotinib resistance in BxPC-3ER cells affected EMT. Immunofluorescence data from BxPC-3ER cells showed enhanced expression of mesenchymal markers, Snail1 and vimentin, and a decrease in expression of the epithelial marker, E-cadherin (Figure 1E). Consistent with this, qRT-PCR (Figure 1F) and western blot analysis (Figure 1G) confirmed the expression of E-cadherin and Snail1, a transcriptional repressor of E-cadherin, in both BxPC-3 and BxPC-3ER cells. Therefore, these data suggest that acquired resistance to erlotinib in BxPC-3ER cells might be associated with attained EMT properties.

### Erlotinib resistance accompanies molecular alterations in BxPC-3ER cells

**Figure 2:**
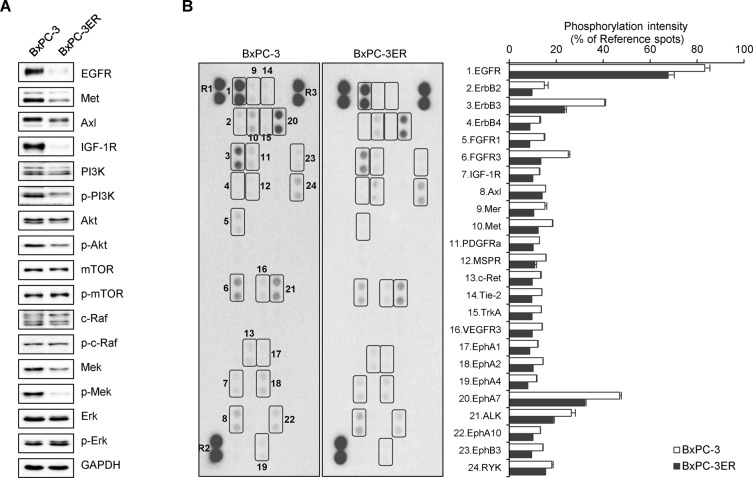
Erlotinib resistance accompanies molecular alterations in BxPC-3ER cells **(A)** Expression of RTKs and downstream signaling molecules. Cell lysates were assayed using western blot with antibodies against RTKs and downstream proteins. GAPDH was used as a loading control. **(B)** Phospho-RTK analysis of BxPC-3 and BxPC-3ER cells. Cell lysates were assayed using a human phospho-RTK array kit. Phosphorylation levels were quantified using ImageJ software and normalized to reference spots (R1, R2 and R3). Spots of interest are boxed and numbered as indicated.

To explore the underlying mechanism of erlotinib resistance in pancreatic BxPC-3ER cells, we used a phospho-RTK array and western blot to determine the phosphorylation status of multiple receptor tyrosine kinases (RTKs) and the expression of downstream proteins. As a result, the expression of most RTKs, such as EGFR, Met, Axl and IGF-1R, was highly reduced in BxPC-3ER cells compared to BxPC-3 cells, as was phosphorylation of downstream kinases such as PI3K-Akt and Ras-Erk (Figure 2A). Moreover, phospho-RTK array data revealed that phosphorylation of most RTKs, including EGFR, Met, and Axl, in BxPC-3ER cells was absent in BxPC-3ER cells (Figure 2B). These results suggest that there might be a no direct relationship between RTK activation and erlotinib resistance in BxPC-3ER cells.

### Untargeted and targeted metabolomic analyses reveal coordinate alteration of metabolism in BxPC-3ER cells

**Figure 3:**
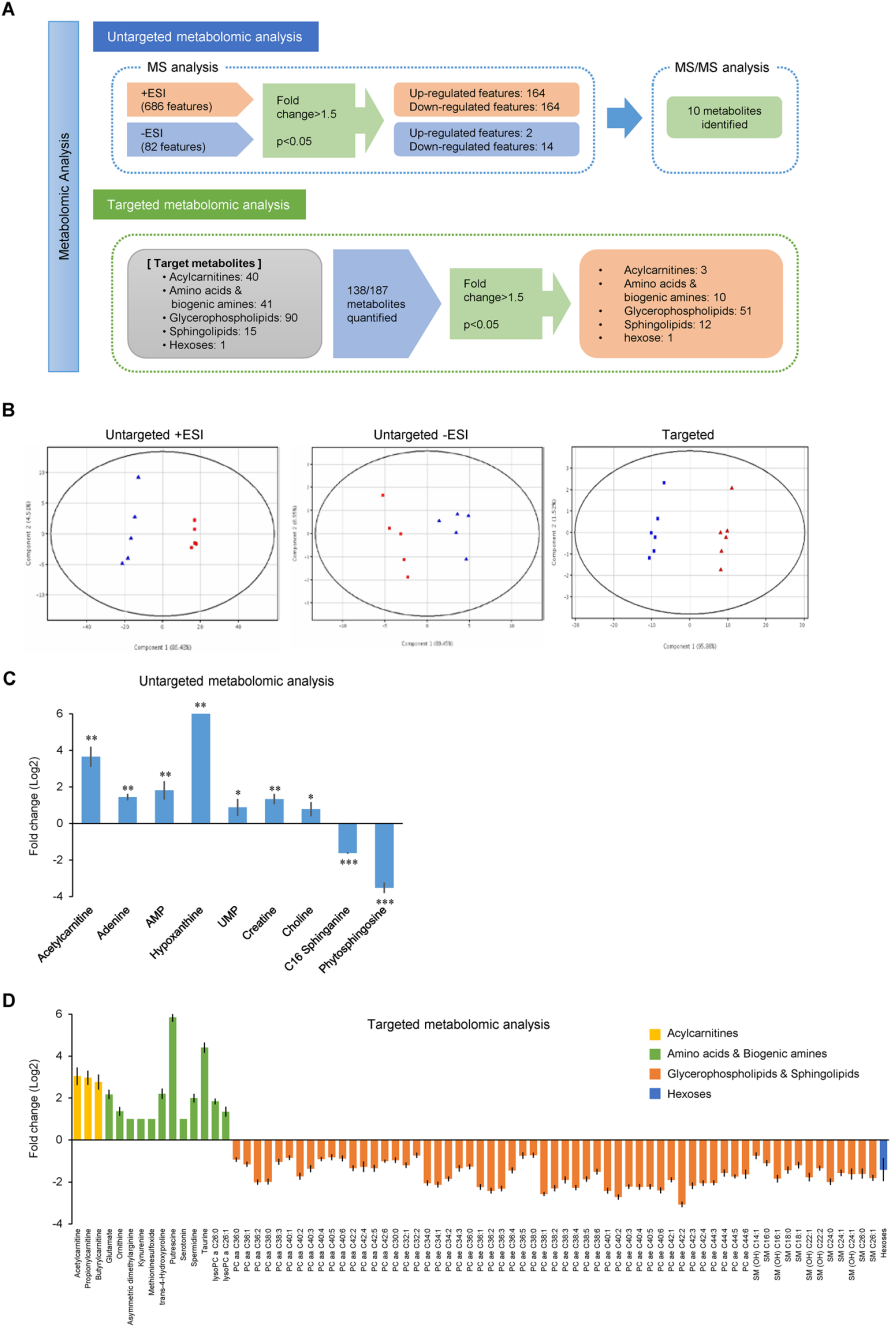
Untargeted and targeted metabolomic analyses reveal coordinate alteration of metabolic pathways in BxPC-3ER cells **(A)** Schematic workflow of untargeted and targeted metabolomic analyses. In untargeted analysis, ion features obtained from MS analysis were examined using a statistical significance analysis and significantly altered ion features were assigned to metabolites by MS/MS analysis. In targeted analysis, target metabolites were quantified, and significantly altered metabolites were selected by statistical significance analysis. **(B)** PCA score plots for metabolites in BxPC-3 (■) and BxPC-3ER (▲) cells. **(C)** Significantly changed metabolites identified in untargeted metabolomics analysis. Error bars show mean ± SD (n = 5). Statistical significance was determined using an un-paired *t*-test and ANOVA with multiple testing correction (Benjamini-Hochberg False Discovery Rate). ^*^p < 0.05; ^**^p < 0.01; ^***^p < 0.001. **(D)** Significantly changed metabolites identified in untargeted metabolomics analysis. Error bars show mean ± SD (n = 6). Un-paired *t*-test and ANOVA with multiple testing correction (Benjamini-Hochberg False Discovery Rate) were used to determine significant differences in quantitative results for selected metabolites, with fold change above 2 and *p*-value below 0.001, between BxPC-3 and BxPC-3ER cells.

Although a decrease in the expression or activity of RTKs could be responsible for the reduced proliferation rate in BxPC-3ER cells, it is necessary to elucidate the cause of the prometastatic phenotype observed in BxPC-3ER cells. To gain insight into the molecular mechanisms that underlie erlotinib resistance in BxPC-3ER cells, we performed untargeted and targeted metabolomics analyses using MS (Figure 3A).

Total compound chromatograms of BxPC-3 and BxPC-3ER cells analyzed using liquid chromatography-hybrid quadrupole time-of-flight mass spectrometry (LC-QTOF-MS) with the positive and negative electrospray ionization (ESI) mode (Supplementary Figure 2A, n = 5) showed acceptable reproducibility. Since one of the six samples produced a higher than 15% deviation in the peak areas of the internal standards, the analytical results from five samples were used for data processing. A total of 686 and 82 ion features, which were deemed satisfactory according to the criteria given in the *Untargeted metabolomic analysis* section, were extracted in the positive and negative ESI mode, respectively, and examined for differential analysis and significance analysis.

The results of principal component analysis (PCA) demonstrated clear clustering between BxPC-3 and BxPC-3ER cells in both ionization modes. This differentiation was mainly described by the first principal component (PC1) which was 86.48% and 89.45% of the observed variations in the positive and negative ESI mode, respectively (first two plots in Figure 3B). Volcano plots showed significantly up- or downregulated ion features in BxPC-3ER cells compared to BxPC-3 cells (fold change > 1.5, *p* < 0.05). In the positive ESI mode, 164 ion features (24% of all features) were upregulated and the same number was downregulated. In the negative ESI mode, 2 ion features (2% of all features) were upregulated and 14 (17%) were downregulated (Supplementary Figure 2B).

Figure 3C shows fold changes in metabolites that were significantly different between BxPC-3 and BxPC-3ER cells, and data from the LC-QTOF-MS analysis, together with *p*-values and log2 fold changes (summarized in Supplementary Table 1). Choline, acetylcarnitine, creatine, adenine, adenosine-5′-monophosphate (AMP), hypoxanthine and uridine-5′-monophosphate (UMP) were upregulated in BxPC-3ER cells, while C16 sphinganine and phytosphingosine were downregulated, compared to BxPC-3 cells. Interestingly, significantly higher levels of the nucleotide AMP, and its related nucleobases, adenine and hypoxanthine, were detected in BxPC-3ER cells compared to BxPC-3 cells. The related nucleoside, adenosine, was also increased in BxPC-3ER cells (log2 fold changes, 0.314; *p* = 0.146) in spite of falling short of the cut-off *p*-value. Adenosine triphosphate was not detected in either cell type, probably due to the lack of sensitivity of the method. Nevertheless, these results imply that activation of adenine metabolism contributes to acquired erlotinib resistance in pancreatic cancer cells.

MS-based targeted metabolomic analysis was performed using a commercial analytical tool for the simultaneous quantification of 187 metabolites, of which 138 were (semi-) quantified (Figure 3A, Supplementary Table 2). The quantitative results were used for further statistical analysis. The PCA score plot (third plot in Figure 3B) revealed that the (semi-) quantitative results of those metabolites showed significant differences between the two groups of cells, mainly by PC1, which was 95.86% of the observed variations (last plot in Figure 3B). The significance analysis selected 77 metabolites (3 acylcarnitines, 10 amino acids and biogenic amines, 51 glycerophospholipids, 12 sphingolipid and hexose) that were significantly different between the two cell types (Figure 3D). *p*-values and log2 fold changes are summarized in Supplementary Table 2. Significant increases in the levels of short-chain acylcarnitines, selected amino acids and biogenic amines and lysophosphatidylcholines, and significant decreases in levels of parts of the phosphatidylcholine and sphingolipid group were observed in BxPC-3ER cells. Three acylcarnitines, acetylcarnitine (C2), propionylcarnitine (C3) and butyrylcarnitine (C4), were present at higher concentrations (range of log2 fold changes; 2.42 ∼ 2.69) in BxPC-3ER cells than in BxPC-3 cells. Of the amino acids and biogenic amines, glutamate, ornithine, asymmetric dimethylarginine (DMA), methionine sulfoxide, putrescine, spermidine, trans-4-hydroxyproline and taurine were upregulated in BxPC-3ER cells, while kynurenine and serotonin were downregulated. Notably, the synthesis of polyamines such as putrescine and spermidine was activated in BxPC-3ER cells, despite no significant change in spermine (data not shown). Moreover, ornithine, the major source for the biosynthesis of those polyamines, was increased in BxPC-3ER cells. The majority of glycerophospholipids and sphingolipids were quantified in both cell types. Among them, two lysophosphatidylcholines (lysoPCs) were increased (log2 fold change; 1.56 and 1.06), and 49 phosphatidylcholines (PCs) were decreased (range of log2 fold changes; -3.37 ∼ -1.04), in BxPC-3ER cells. Twelve out of 14 sphingomyelins (SMs) were also downregulated (range of log2 fold changes; -1.06 ∼ -2.30) in BxPC-3ER cells. Thus, our targeted metabolomic analysis of BxPC-3 and BxPC-3ER cells revealed that alterations in phospholipid turnover and polyamine metabolism accompany the acquisition of resistance to erlotinib in BxPC-3 cells.

### Transcriptome profiling reveals coordinated alteration of polyamine metabolism in BxPC-3ER cells

**Figure 4:**
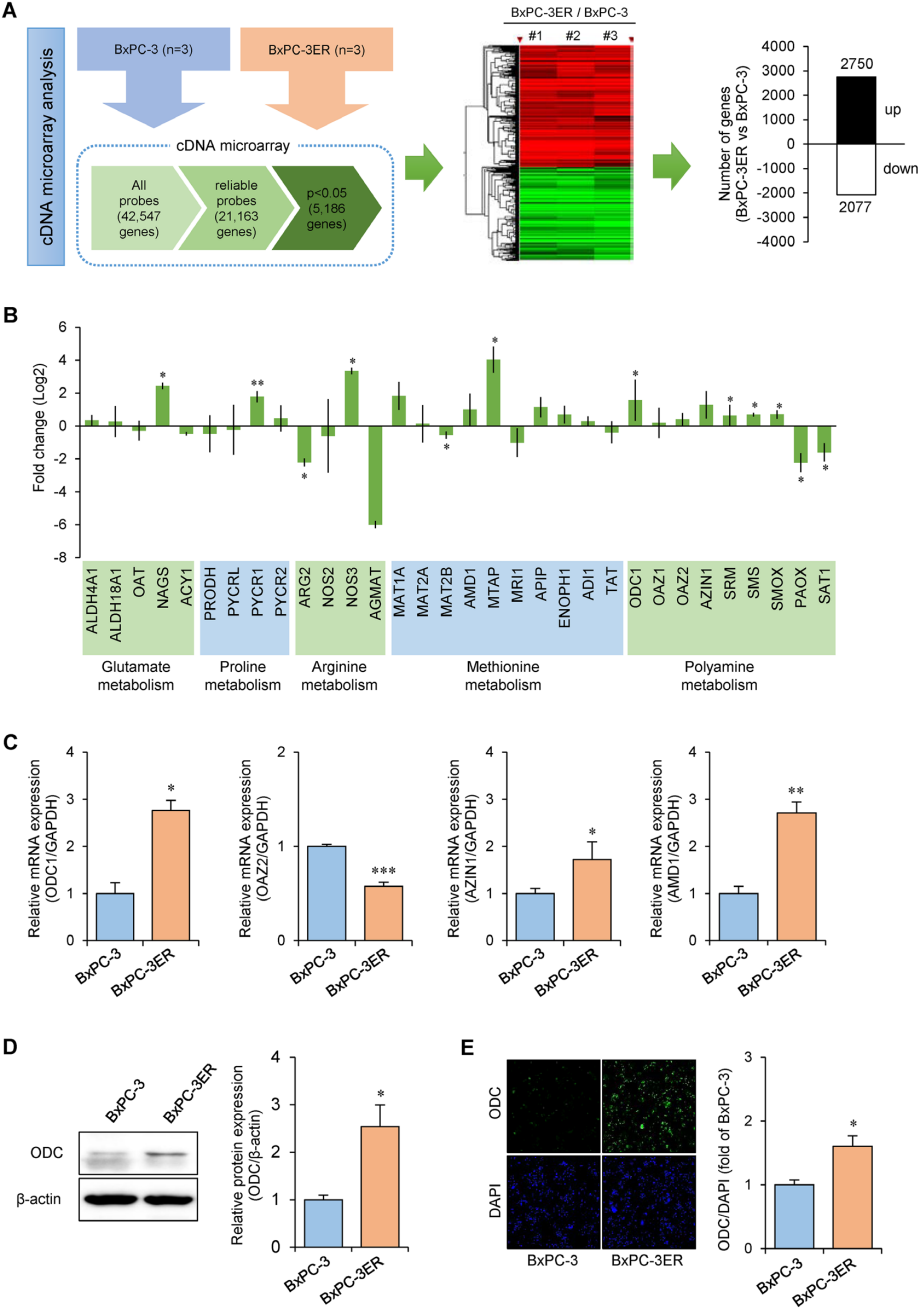
Alteration of polyamine pathway in erlotinib-resistant BxPC-3ER cells **(A)** Schematic workflow of cDNA microarray analysis. RNA extraction from BxPC-3 and BxPC-3ER cells, sample preparation, and data acquisition by microarray, followed by statistical analysis. **(B)** Relative mRNA expression of several metabolic pathways identified by microarray. Error bars show mean ± SD (n = 3). Statistical significance was determined using a Student's *t*-test. ^*^p < 0.05; ^**^p < 0.01. **(C)** Real-time PCR analysis of polyamine pathway genes. Error bars show mean ± SD (n = 4). Statistical significance was determined using a Student's *t*-test. ^*^p < 0.05; ^**^p < 0.01; ^***^p < 0.001. **(D)** Western blot for ODC expression. Error bars show mean ± SD (n = 4). Statistical significance was determined using a Student's *t*-test. ^*^p < 0.05. **(E)** Immunostaining for ODC expression. Statistical significance was determined using a Student's *t*-test. ^*^p < 0.05.

To examine transcriptomic changes after the acquisition of erlotinib resistance, we conducted a cDNA microarray analysis of BxPC-3 and BxPC-3ER cells. We refined the lists of genes with differential expression between cells using criteria of fold change > 1.5 and *p* < 0.05 as the cutoff. The data identified a total of 4,827 genes that were differentially expressed between the two cell lines. Of these, 2,750 were found to be upregulated and 2,077 were downregulated in BxPC-3ER cells (Figure 4A). Based on our metabolomic profiling data, we then mined the transcriptome profiles to explore polyamine metabolism. Consistent with our metabolomic data, alterations were observed in the metabolism of several amino acids that drive polyamine synthesis (Figure 4B). Interestingly, most polyamine prosynthetic enzymes were upregulated in BxPC-3ER cells, while most polyamine catabolic enzymes were downregulated (Figure 4B). Notably, ornithine decarboxylase (ODC), a rate limiting enzyme in polyamine biosynthesis, was significantly upregulated, whereas ODC antizyme 2 (OAZ2), which targets ODC for proteasomal degradation, was downregulated. Moreover, ODC antizyme inhibitor 1 (AZIN1), which inhibits the activity of OAZ2, was upregulated in BxPC-3ER cells, promoting ODC activity in erlotinib-resistant pancreatic cells. To validate the microarray data, we confirmed the changes in gene expression by qRT-PCR (Figure 4C). While at the protein level, the significant increase in ODC expression in BxPC-3ER cells was confirmed by western blot (Figure 4D) and immunocytochemistry (Figure 4E). In summary, our transcriptomic analysis revealed changes in the expression of key enzymes that regulate polyamine metabolism that are consistent with alterations in metabolites in the polyamine pathway, as revealed by the metabolomics analysis.

### ODC-dependent putrescine confers erlotinib resistance to BxPC-3ER cells

**Figure 5:**
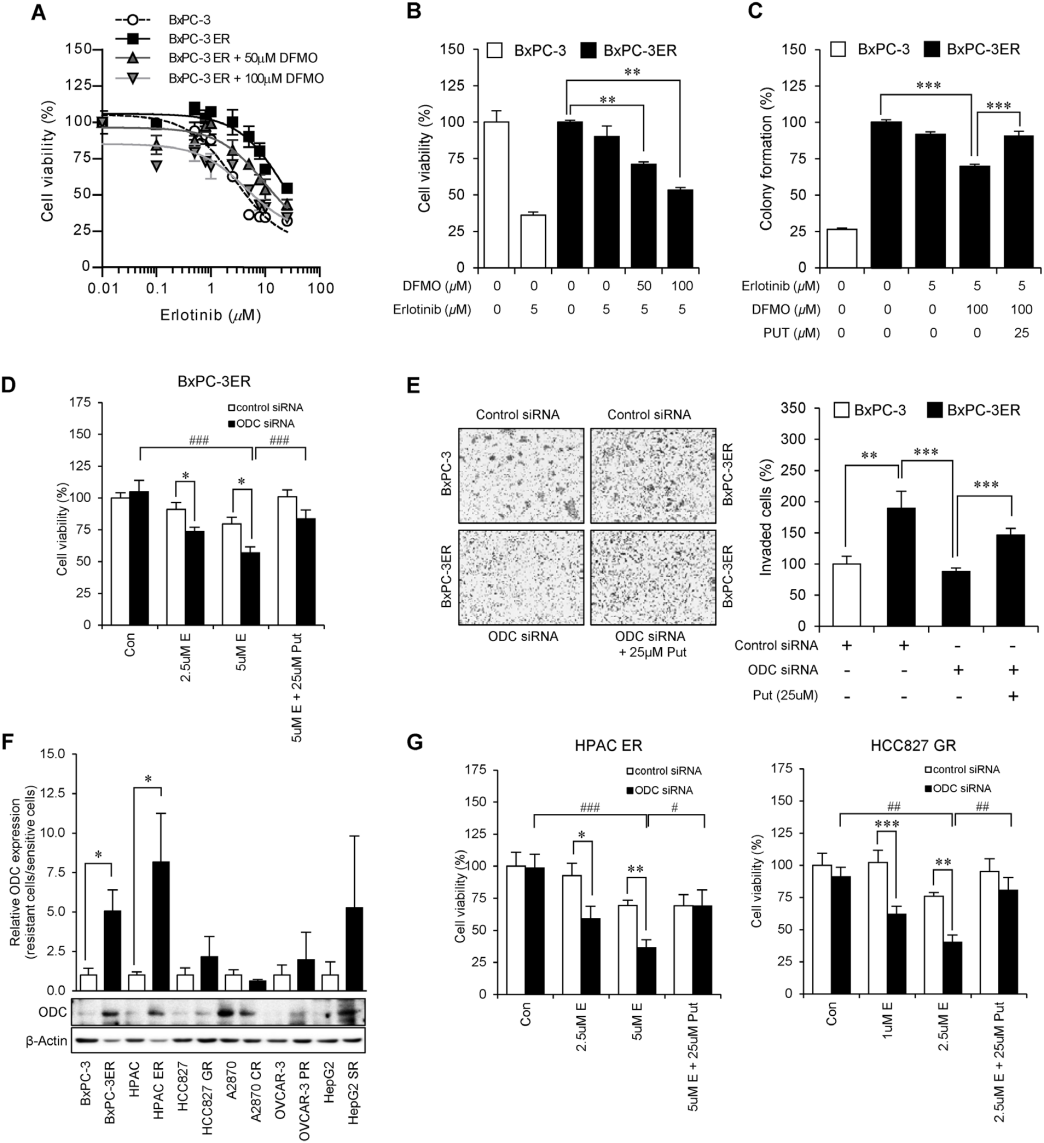
Putrescine confers erlotinib-resistance to BxPC-3ER cells **(A)** ODC inhibition by DFMO restores sensitivity to erlotinib in BxPC-3ER cells. Cell viability of BxPC-3 and BxPC-3ER cells after incubation with serial dilutions of erlotinib was determined using an MTT assay. Statistical analysis performed using two-way ANOVA (*p* < 0.001). Error bars represent mean ± SD (n = 4). **(B)** Dose-dependent DFMO treatment overcame erlotinib resistance in BxPC-3ER cells. Error bars represent mean ± SD (n = 4). ^**^p < 0.01. **(C)** Colony formation assay. Cells were treated as indicated and followed for 7 days. Statistical significance was determined using a Student's *t*-test. ^***^p < 0.001. **(D)** ODC-dependent putrescine confers resistance to erlotinib in BxPC-3ER cells. Viability of BxPC-3ER cells transfected with control or ODC siRNAs was determined using an MTT assay. Error bars represent mean ± SD (n = 4). Statistical significance was determined using a Student's *t*-test. ^*^p < 0.05, ^###^*p* < 0.001. **(E)** Putrescine treatment enhances invasiveness of BxPC-3ER cells. Statistical analysis was conducted using a Student's *t*-test. ^**^p < 0.01, ^***^p < 0.001. Error bars show mean ± SD (n = 5). **(F)** Relative ODC expression in drug-resistant cells to several anti-cancer therapeutics. Western blot analysis was carried out using erlotinib-resistant pancreatic (BxPC-3ER, HPAC-ER), gefitinib-resistant non-small cell lung (HCC827-GR), cisplatin- or paclitaxel-resistant ovarian (A2780-CR, OVCAR-3PR), sorafenib-resistant liver (HepG2-SR) cancer cells and their counterparts. **(G)** ODC-dependent putrescine confers resistance to erlotinib in HAPC-ER cells and gefitinib in HCC827-GR cells. Viability of HPAC-ER and HCC827-GR cells treated with control or ODC siRNAs was determined using an MTT assay. Error bars represent mean ± SD (n = 4). Statistical significance was determined using a Student's *t*-test. ^*^p < 0.05, ^**^p < 0.01, ^##^*p* < 0.01, ^###^*p* < 0.001.

Polyamines have been implicated in various human cancers, likely due to their important role in cancer cell growth [13]. Recently, it has been suggested that ODC-dependent putrescine confers resistance to 5-fluorouracil (5-FU) in colon cancers, implying a possible role for ODC and polyamines in drug resistance [14]. Based on these and our own findings, we hypothesized that ODC-dependent polyamines could be key regulators of acquired erlotinib resistance in BxPC-3ER cells. To test this, we added an irreversible ODC inhibitor, difluoromethylornithine (DFMO) to BxPC-3ER cells and assessed whether the cells could overcome drug resistance to erlotinib. Figure 5A shows that treatment with DFMO restored sensitivity to erlotinib in BxPC-3ER cells, to levels comparable with those for erlotinib-sensitive BxPC-3 cells. More precisely, treatment with 100 μM DFMO in the presence of erlotinib reduced cell viability to below 50% in an MTT assay (Figure 5B).

ODC is known to be a rate-limiting enzyme in the biosynthesis of polyamines such as putrescine, spermidine, and spermine. We found that cellular levels of polyamines were highly maintained in BxPC-3ER cells, which correlates with ODC expression levels. We then investigated whether putrescine could recover colony forming capacity in DFMO-treated BxPC-3ER cells. Consistent with cell viability data, our results revealed that treatment of BxPC-3ER cells with 100 μM DFMO in the presence of erlotinib resulted in a 40% reduction in colony formation. However, treatment with putrescine recovered the colony forming ability of these cells up to 90%, even in the presence of DFMO and erlotinib (Figure 5C). Next, we verified that knockdown of ODC increased the sensitivity of BxPC-3ER cells to erlotinib, and putrescine treatment conferred resistance to BxPC-3ER cells (Supplementary Figure 3 and Figure 5D). Using an invasion assay, we also verified that ODC-dependent putrescine is involved in the enhanced invasiveness of BxPC-3ER cells (Figure 5E). We next examined whether increased ODC expression is relevant in other cancer cell lines that exhibit resistance to various therapeutics. Of the 6 kinds of drug-resistant cancer cells we tested, 5 exhibited higher levels of ODC expression levels compared to their parental cells (Figure 5F). Additionally, we verified the desensitizing effect of putrescine on erlotinib or gefitinib treatment in other erlotinib-resistant pancreatic cell lines, HPAC-ER and gefitinib-resistant non-small cell lung cancer cells, HCC827-GR (Figure 5G). Therefore, these results suggest that ODC-dependent putrescine might confer drug resistance in several cancer cells as well as BxPC-3ER cells.

## DISCUSSION

Multi-omics approaches, the high-throughput analyses of multiple omics data, can provide insights into comprehensive changes in biological pathways induced by external stimuli or genetic alterations. Moreover, the results can be used to identify markers of the disease process [15]. In particular, metabolomics is a useful tool to bridge the gap between a phenotype and a related biological regulator [16–18]. In the present study, the metabolic changes that accompany the acquisition of resistance to erlotinib in BxPC-3 cells were comprehensively investigated using targeted and untargeted metabolomic analyses. In the untargeted analysis, overall patterns of potential metabolites were investigated for differences between biological samples and the discovery of differing metabolites. [19–24]. On the other hand, the targeted analysis was based on the quantification of a group of selected metabolites, followed by statistical analysis, and provided insight into the functional role of specific metabolites and related regulators. However, depending on the selection of metabolites used in the analysis, broadening our knowledge of metabolite changes could be limited and/or finding novel metabolites could be almost impossible [19, 25]. In the present study, integrated results from the untargeted and targeted metabolomic analyses revealed that the acquisition of erlotinib resistance in BxPC-3 cells causes multilateral changes in tumor metabolism. Notably, it highlighted the activation of adenine metabolism, polyamine metabolism, lipid metabolism and possibly fatty acid metabolism during this process.

The significant increase in levels of short-chain acylcarnitines observed in our results could indicate defects in beta-oxidation [26–28] as a result of acquisition of erlotinib resistance. The increase in lysoPCs and decrease in PCs suggest that phospholipase A_2_ is being activated in BxPC-3ER cells. Low levels of PCs could further affect biosynthesis of SMs, which are a component of the animal cell membrane and are related to the regulation of transmembrane signaling, since PCs are substrates of SM synthase [29]. The composition of cancer cell membrane lipids, including SMs and PCs, can affect drug transportation into cells. It was previously reported that acquired drug resistance can change lipid biosynthesis and the composition of membrane lipids and, consequently, reduce drug influx in drug-resistant cancer cells [30]. The acquisition of erlotinib resistance in BxPC-3 cells could therefore also alter cell membrane lipids and prevent further transport of erlotinib into the cells.

**Figure 6:**
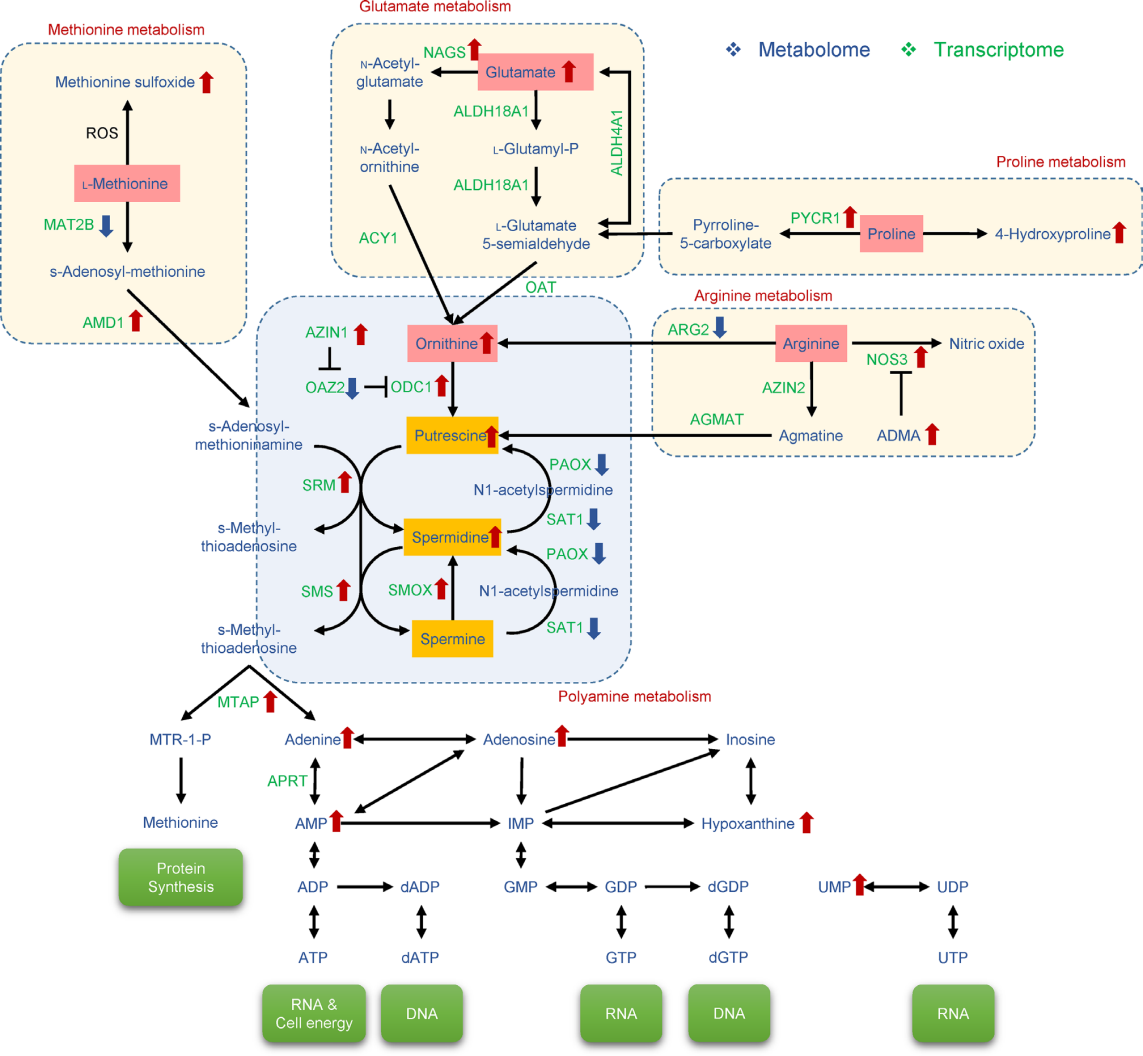
Metabolic pathways implicated in erlotinib resistance in BxPC-3ER cells Cooperative regulation of polyamine metabolism by methionine, glutamate, proline, and arginine pathways. Polyamines (putrescine, spermidine, spermine) are synthesized from ornithine by ornithine decarboxylase (ODC). Core metabolites and enzymes of depicted pathways are shown. AMD, S-adenosylmethionine decarboxylase; SRM, spermidine synthase; SMS, spermine synthetase; SAT, spermine/spermidine-N-acetyltransferase; PAOX, polyamine oxidase; SMOX, spermine oxidase; OAZ, ODC antizyme; AZIN, antizyme inhibitor.

Dysregulation of polyamines was previously reported in relation to gene expression, cell proliferation, cellular stress, and several human diseases, including cancer. Higher concentrations of polyamines were observed in biological samples from many cancer patients, compared to healthy people [31]. Adenine metabolism and polyamine metabolism are closely related, since an intermediate product in polyamine metabolism, methylthioadenosine (MTA), produces adenine to activate the methionine cycle [32]. Therefore, polyamine and methionine metabolism could be altered in BxPC-3ER cells, as shown in Figure 6. MTA levels were not significantly different between the two types of cells; however, MTA was downregulated in the media of BxPC-3ER cells (*p* = 4.8e^−9^, log2 fold change = -5.7), compared to BxPC-3 cells (our unpublished results).

Our findings elucidate mechanisms of acquired resistance to erlotinib and identify potential candidates for treatment of pancreatic cancers. One of the possible mechanisms for acquired resistance to EGFR-TKIs can be explained by the occurrence of secondary mutations in the EGFR gene [33] and activation of redundant receptor proteins such as Met [34] and Axl [35] in several human cancers. In this context, we hypothesized that activation of alternative RTKs to compensate for the loss of EGFR dependence might result in the conversion of BxPC-3 to BxPC-3ER cells in response to erlotinib treatment. Unexpectedly, however, the expression and phosphorylation of most RTKs, such as EGFR, Met, Axl and IGF-1R, was highly reduced in BxPC-3ER cells measured by western blot and phospho-RTK array analyses. These results were not coincided with previous reports [33–35], which gave us a clue to find other possible candidates that responsible for erlotinib resistance in BxPC-3ER cells. To do this, we performed a transcriptomic analysis to begin to elucidate the possible mechanism of erlotinib-resistance in pancreatic cancer cells. Of interest, our multi-omics analyses identified changes in cellular metabolism in terms of dynamic changes in metabolites and their responsible genes in erlotinib-resistant pancreatic BxPC-3ER cells. One of the most notable findings was that polyamine metabolic pathways were considerably dysregulated in erlotinib-resistant BxPC-3ER cells. Polyamines, including putrescine, spermidine and spermine, are polycations that are required for eukaryotic cell growth and differentiation [31]. Several lines of evidence have suggested that increased polyamine levels are associated with increased cell growth, tumor invasion and metastasis, whereas polyamine depletion leads to cell cycle arrest in cancer cells [36]. Intracellular levels of polyamines are tightly controlled by multi-step reactions, mainly performed by ODC and S-adenosylmethionine decarboxylase (AMD). ODC, a rate limiting enzyme of polyamine biosynthesis, converts ornithine to putrescine, which is sequentially converted to spermidine and spermine by other enzymes, spermidine synthase (SRM) and spermine synthetase (SMS), respectively. The polyamine catabolic pathway is regulated by central enzymes, including spermine/spermidine-N-acetyltransferase (SAT), polyamine oxidase (PAOX) and spermine oxidase (SMOX). ODC is tightly regulated by ODC antizyme (OAZ) and antizyme inhibitor (AZIN) in the polyamine pathway. OAZ effectively controls polyamine levels by mediating the inactivation and degradation of ODC. AZIN plays a role in the regulation of OAZ. Collectively, our metabolomic analysis confirmed that ODC-dependent polyamine levels, such as putrescine and spermidine, were much higher in BxPC-3ER cells than in parental BxPC-3 cells. Also, our microarray analysis verified that the expression of enzymes responsible for polyamine biosynthesis was consistent with the increase in polyamine synthesis.

Few studies have focused on the role of polyamines in cancer therapeutics, and yet understanding polyamine function and metabolic regulation in human cancers is becoming increasingly important for the development of anti-cancer drugs [36, 37]. A recent study suggested that the polyamine pathway may be a crucial target for the prevention of 5-FU-induced chemoresistance in colorectal cancer cells [14]. In this paper, the authors clearly show that putrescine induces resistance to 5-FU treatment by antagonizing the JNK-caspase-3 pathway, implying an important role for putrescine in regulating resistance to cancer therapeutics [14]. Consistent with this, our data suggest that activation of the ODC-dependent polyamine pathway is responsible for the acquisition of erlotinib resistance in pancreatic cancer cells. We demonstrated that depletion of polyamines using the ODC inhibitor, DFMO, or knockdown of ODC using siRNA during erlotinib treatment increases sensitivity to erlotinib in BxPC-3ER cells, which could be reversed by putrescine treatment. Therefore, our study shows that ODC-dependent polyamines are involved in the acquisition of erlotinib resistance, and that high expression and activity of ODC correlates with desensitization to erlotinib treatment in cancer drug-resistant cells.

Collectively, by investigating metabolic profiles between erlotinib-sensitive and -resistant human pancreatic cancer cells, our multi-omics approaches have identified a mechanism for erlotinib resistance. We suggest that an increase in putrescine levels is one of the leading mechanisms underlying the acquisition of erlotinib resistance in human pancreatic cancers, and thereby propose that targeting ODC, a key enzyme in the polyamine pathway, might be a novel strategy for overcoming resistance to erlotinib.

## MATERIALS AND METHODS

### Chemicals and reagents

Erlotinib was obtained from LC Laboratories (Woburn, MA, USA). 3-(4,5-Dimethylthiazol-2 yl)-2,5-diphenyltetrazolium bromide (MTT), formic acid, potassium chloride, potassium phosphate monobasic, sodium chloride, sodium phosphate dibasic, 2-aminoanthracene and 2,3,4,6-pentafluorobenzoic acid were purchased from Sigma-Aldrich (St. Louis, MO, USA). AbsoluteIDQ^®^ p180 kit was obtained from Biocrates Life Sciences AG (Innsbruck, Austria).

### Cell lines and cell culture

Human pancreatic adenocarcinoma BxPC-3 cell line was obtained from the American Type Culture Collection (Manassas, VA, USA) and maintained in RPMI-1640 medium with L-glutamine supplemented with 10% FBS (Gibco-BRL; Grand Island, NY, USA) at 37°C in a humid incubator with 5% CO_2_. BxPC-3ER cells were generated by continuous exposure of parental BxPC-3 cells to erlotinib for > 6 months.

### Untargeted metabolomic analysis

Cells were prepared using a method described elsewhere [38, 39], with some modification. Adherent cells were quickly washed with phosphate buffered saline (PBS), quenched with ice-cold 0.9% (w/v) sodium chloride solution and centrifuged to collect cell pellets (1,000 g, 5 min, 4°C). One milliliter of a mixed solution of acetonitrile, methanol, and water (40:40:20, v/v/v) was added to cell pellets. 2-aminoanthracene (10 μL of 100 μg/mL) and 2,3,4,6-pentafluorobenzoic acid (5 μL of 1 mg/mL) were used as internal standards for ESI in positive and negative mode, respectively, in LC-QTOF-MS. Metabolites were extracted by shaking the samples for 1 h at 4°C. After centrifugation (13,000 g, 10 min, 4°C), the supernatant was evaporated under a stream of nitrogen at 40°C. The residue was reconstituted in 50 μL acetonitrile and mobile phase A solution (1:9, v/v).

LC-QTOF-MS analysis was performed using an Agilent 6530 Accurate-Mass Q-TOF LC/MS System with Agilent 1290 Infinity LC (Agilent Technologies, Santa Clara, CA, USA). Zorbax SB-C8 (3.5 μm, 2.1×30 mm, Agilent Technologies) and Zorbax SB-Aq (1.8 μm, 2.1×100 mm, Agilent Technologies) were used for guard and analytical columns, respectively, and maintained at 40°C. Mobile phases consisted of 0.1% formic acid in water (A) and 0.1% formic acid in acetonitrile (B). Gradient conditions were as follows: 0-30 min, 1-20% B; 30-40 min, 20-90% B; 40-45 min, 90% B; 45-47 min, 90-1% B; 47-52 min 1% B at a flow rate of 400 μL/min.

The MS system was operated using ESI in both positive and negative ionization modes. Optimized conditions of the QTOF-MS system were as follows: drying gas temperature, 300°C; drying gas flow, 10 L/min; nebulization pressure, 45 psi; sheath gas temperature, 350°C; sheath gas flow, 10 L/min; capillary voltage, 3500 V; nozzle voltage, 0 V; fragmentor voltage, 175 V; skimmer voltage, 65 V. Mass range was 50-1700 m/z and scan rate was 2.00 spetra/sec for both MS and MS/MS analyses. Purine (exact mass for [M+H]^+^ = 121.050873) and 1,2,3,4,5,6-hexakis(2,2,3,3-tetrafluoropropoxy)-1,3,5,2,4,6-triazatriphosphinane (exact mass for [M+H]^+^ = 922.009798) were used for mass calibration. MS/MS analysis was performed using three different collision energy values; 10, 20 and 40 eV.

Data from MS runs were processed using a MassHunter Profinder (version B.06.00) and Mass Profiler Professional (MPP, version B.13.1) software (Agilent Technologies). Metabolic differences between BxPC-3 and BxPC-3ER cells were evaluated by PCA followed by an un-paired *t*-test and analysis of variance (ANOVA) with multiple testing correction (Benjamini-Hochberg False Discovery Rate). Quantitative results were normalized to protein concentrations, which were determined using a Bicinchoninic Acid (BCA) protein assay kit (Thermo Fisher Scientific, Rockford, IL, USA).

Tentative identification of significantly altered features was performed by comparing their accurate masses with those in the MassHunter METLIN Metabolite Personal Compound Database and Library (Demo version, summer, 2014). From the list of tentative metabolites, those with matching scores above 80 were rigorously inspected to confirm identification either by comparison of chemical standards or by MS/MS analysis. The MS/MS fragmentation spectra of metabolites in cells obtained at collision energy levels of 10, 20 and 40 eV were compared to those in the library. In cases of no library matching, the MS/MS spectra were structurally investigated and confirmed by Molecular Structure Correlator (MSC) software (Agilent Technologies).

### Targeted metabolomic analysis

MS-based targeted metabolomic analysis was performed using an Absolute*IDQ* p180 kit (Biocrates Life Sciences AG, Innsbruck, Austria), which allowed simultaneous quantification of 187 metabolites (40 acylcarnitines, 41 amino acids and biogenic amines, 90 glycerophospholipids, 15 sphingolipids and a sum of hexoses including glucose). Cell culture lysates were prepared using a modified extraction protocol, provided by the manufacturer [40]. After media was removed, BxPC-3 and BxPC-3ER cells were washed twice with ice-cold PBS and lysed in 10 mM phosphate buffer. Three cycles of sonication (15 sec) followed by freeze (liquid nitrogen for 30 sec) and thaw (instantly in 98°C heat block) processing were performed, and samples were finally centrifuged to collect cell lysates (20,000 g, 10 min, 4°C), which were loaded into the kit.

An AB Sciex 4000 QTrap mass spectrometer (Sciex, Framingham, MA, USA) in the multiple reaction monitoring detection mode with ESI was used for analysis at Inha University Hospital Clinical Trial Center (Incheon, Korea). Samples were injected to MS by flow injection analysis for the determination of amino acids and biogenic amines and by LC for the determination of other groups. The kit was validated using MetValTM (Biocrates Life Sciences AG) software and analytical results were processed using AnalystTM (Sciex) and MetValTM software.

Un-paired *t*-test and ANOVA with multiple testing correction (Benjamini-Hochberg False Discovery Rate) were used to determine significant differences of the quantitative results of selected metabolites, with criteria of fold change higher than 2 and *p*-value below 0.001, between the two types of cells. Quantitative results were normalized by protein concentration using a BCA protein assay kit.

### cDNA microarray analysis

An Agilent Human Whole-genome expression microarray (V2) (Agilent Technologies, Inc., Palo Alto, CA. USA) was used for the microarray analysis. Total RNA was isolated from BxPC-3 and BxPC-3ER cells using TRIzol^TM^ reagent (Invitrogen, USA), and RNA integrity was measured using a Bioanalyzer 2100 RNA Nano kit (Agilent Technologies). For control and test RNAs, the synthesis of target cRNA probes and hybridization were performed using a Low RNA Input Linear Amplification kit PLUS (Agilent Technologies) according to the manufacturer's instructions. Briefly, 1 μg each total RNA sample and T7 promoter primer mix were mixed and incubated at 65°C for 10 min. cDNA master mix (5× first strand buffer, 0.1 M DTT, 10 mM dNTP mix, RNase-Out, and MMLV-RT) was prepared and added to the RNA-primer mixture. Samples were incubated at 40°C for 2 h for reverse-transcription and double-strand cDNA (dsDNA) synthesis and terminated by incubating at 65°C for 15 min. The transcription master mix was prepared according to the manufacturer's protocol (4× Transcription buffer, 0.1 M DTT, NTP mix, 50% PEG, RNase-Out, Inorganic pyrophosphatase, T7-RNA polymerase, and Cyanine 3/5-CTP), added to the dsDNA reaction mixture and incubated at 40°C for 2 h for transcription of dsDNA. During transcription-amplification, control and test cRNAs were labeled with Cy3-CTP and Cy5-CTP, respectively. Amplified and labeled cRNA Awas purified and quantified using a ND-1000 spectrophotometer (NanoDrop Technologies, Inc., Wilmington, DE) to check labeling efficiency. Fragmentation of cRNA was performed by adding 10× blocking agent and 25× fragmentation buffer and incubating at 60°C for 30 min. Fragmented cRNA was resuspended in 2× hybridization buffer and directly pipetted onto a microarray placed in a Hybridization Chamber (Agilent Technologies). Incubating the hybridization chamber at 42°C for 16 h with mild agitation, allowed competitive hybridization reactions to occur between labeled targets and probes on the microarray. To eliminate the non-specific binding, hybridized microarrays were washed with a Gene Expression Wash Buffer Kit (Agilent Technologies). Finally, microarrays were spin-dried and stored in the dark until scanning.

Scanned images were analyzed using a Feature Extraction program (Agilent Technologies). Average fluorescence intensity for each spot was calculated and local background was subtracted. All data manipulation and selection of fold changed genes were performed using GeneSpring 7.3.1 (Agilent Technologies). All numerical data were normalized as follows, (1) data transformation: set measurements less than or equal to 0.01, (2) per chip: normalize to 50^th^ percentile, (3) per gene: normalize to median.

### Cell viability assay

Cell viability was measured using an MTT assay. Briefly, at the end of treatment, media was replaced with fresh RPMI-1640 medium supplemented with MTT (0.5 mg/mL MTT; 100 μL/well) and incubated at 37°C for 4 h. After aspiration, 100 μL dimethyl sulfoxide (DMSO) was added and plates were agitated for 3 min. Absorbance at 565 nm was read using a Tecan Infinite^®^ F200 PRO plate reader (Promega Corporation, Madison, WI, USA).

### Western blot analysis

After appropriate treatments, cells were lysed in ice-cold lysis buffer (50 mM Tris-HCl pH 8.0, 150 mM NaCl, 1% NP40) and equal amounts of protein lysate were separated using SDS-PAGE and transferred to a PVDF membrane (Bio-Rad, Hercules, NJ). Membranes were blocked with 5% skim milk in TBS-T, then incubated with primary antibodies. After incubation with species-specific horseradish peroxidase-conjugated secondary antibodies, proteins were visualized using SuperSignal^®^ West Dura Extended Duration Substrate (Thermo Scientific, Waltham, MA) and developed with LAS-3000 (Fuji, Japan) according to the manufacturer's instructions.

### Soft agar colony formation assay

BxPC-3 or BxPC-3ER cells (1×10^4^ cells/well) were suspended in Basal Medium Eagle (BME; 1 mL with 10% FBS and 0.33% bacto agar) and plated onto a layer of solidified agar (BME with 10% FBS and 0.5% bacto agar). After incubation at 37°C with 5% CO_2_ for 7 days, colonies were observed using a light microscope (magnification, x40).

### Invasion assay

A cell invasion assay was performed using a BD BioCoat Matrigel Invasion Chamber. Cells were suspended in culture media (1×10^5^ cells/mL) and 0.5 mL cell suspension (5×10^4^ cells/mL) was added to the upper chambers. After incubation for 48 h, media was carefully removed, and membranes containing cells on the lower surface were fixed with methanol and stained with H&E. The upper surface of membrane gently scrubbed with a cotton swab to remove cells. Stained cells were quantified under a light microscope.

### Immunocytochemistry

Cells were plated onto submerged glass coverslips and incubated for 24 h. Cells were fixed with 3.7% formaldehyde for 15 min and permeabilized with 0.1% Triton X-100 (in DPBS) for 10 min at room temperature (RT). After fixation and permeabilization, cells were incubated overnight at 4°C with primary antibodies to detect Snail1 (Santa Cruz, CA, USA), E-Cadherin (Cell Signaling Technology, Beverly, MA), Vimentin (Cell Signaling Technology, Beverly, MA), or ODC (Santa Cruz, CA, USA). Cells were rinsed with 0.1% DPBS-T, and incubated with an Alexa 488-conjugated secondary antibody for 1 h at RT in the dark. Nuclei were counterstained with 0.1 μg/mL 4′,6-diamidino-2-phenylindole (DAPI). Images were obtained using a fluorescence microscope.

### RNA interference

Double-stranded siRNAs against human *ODC1* (NM_002539.1) were synthesized by Bioneer (Daejeon, Republic of Korea). All siRNAs contained symmetric 3′ overhangs of two deoxythymidines. Specific siRNA sequences for human *ODC1* were as follows: *ODC1*_#1 siRNA, 5′-CGAGUGAGCAGACCUUUAU(dTdT)-3′; *ODC1*_#2 siRNA, 5′-CGACGAUCUACUAUGUGAU(dTdT)-3′; *ODC1*_#3 siRNA, 5′-GAAACAUCUGAGGUGGUUA(dTdT)-3′. Negative control siRNA was obtained from Bioneer (Cat. No.: SN-1003). BxPC-3 and BxPC-3ER cells were transfected with 100 pM/6-well or 50 pM/96-well siRNAs using Oligofectamine reagent (Invitrogen) according to the supplier's protocol. After transfection, cells were used for western blot analysis or cell viability assay.

### Real-time RT-PCR analysis

Total RNA was isolated from BxPC-3 and BxPC-3ER cells using TRIzol^TM^ reagent (Invitrogen, USA) according to the manufacturer's instructions. First-strand cDNA was synthesized from 2 μg total RNA using M-MLV reverse transcriptase (Promega, Madison, WI, USA). Real-time PCR of selected genes was performed using double-stranded DNA binding dye SYBR Green and a LightCycler480 (Roche). Forward and reverse primers used in real-time PCR analyses were as follows: *Snail1*, 5′-GAGGCGGTGGCAGACTAG-3′ and 5′-GACAC ATCGGTCAGACCAG-3′; *E-Cadherin*, 5′-TGCCCA GAAAATGAAAAAGG-3′ and 5′-GGATGACACAGC GTGAGAGA-3′; *ODC1*, 5′-ATGGCTTCCAGAGGCC GAC-3′ and 5′-TTGCTGCATGAGTTGCCACGCA-3′; *OAZ2*, 5′-GTAACTGTCCCCAGCTCCAG-3′ and 5′-ATC TTCGACAGTGGGTGAGG-3′; *AZIN1*, 5′-CTTTCCA TGAACCATCTGCT-3′ and 5′-TTCCAGCATCTTGC ATCTCA-3′; *AMD1*, 5′-GGTGATGGAAGCTGCACA TTT-3′ and 5′-GGTGGTACCACATGTCTTCAA-3′; *GAPDH*, 5′-TCGACAGTCAGCCGCATCT-3′ and 5′-CC GTTGACTCCGACCTTCA-3′. Three replicates were run for each gene. Expression levels of target genes were normalized to GAPDH expression.

### Statistical analysis

Statistical analysis for the results from biological experiments was carried out using a Student's *t*-test or two-way ANOVA using GraphPad Prism5. All data are presented as mean ± SD. *p* < 0.05 was considered statistically significant.

## SUPPLEMENTARY MATERIALS FIGURES AND TABLES



## Abbreviations

ODC: ornithine decarboxylase
ESI: electrospray ionization
LC-QTOF-MS: liquid chromatography-hybrid quadrupole time-of-flight mass spectrometry
PCA: principal component analysis
BCA: Bicinchoninic Acid
MSC: Molecular Structure Correlator
DFMO: difluoromethylornithine
RTK: receptor tyrosine kinase
5-FU: 5-fluorouracil
lysoPCs: lysophosphatidylcholines
SMs: sphingomyelins
