# Supplementary material for: Multi-omics analysis reveals that ornithine decarboxylase contributes to erlotinib resistance in pancreatic cancer cells
Source: Oncotarget. 2017 Oct 6;8(54):92727–42. doi: 10.18632/oncotarget.21572 (PMC5696217; doi:10.18632/oncotarget.21572)
Supplement: Supplementary file 1 [file oncotarget-08-92727-s001.pdf]

Multi-omics analysis reveals that ornithine decarboxylase contributes to erlotinib resistance in pancreatic cancer cells

SUPPLEMENTARY MATERIALS

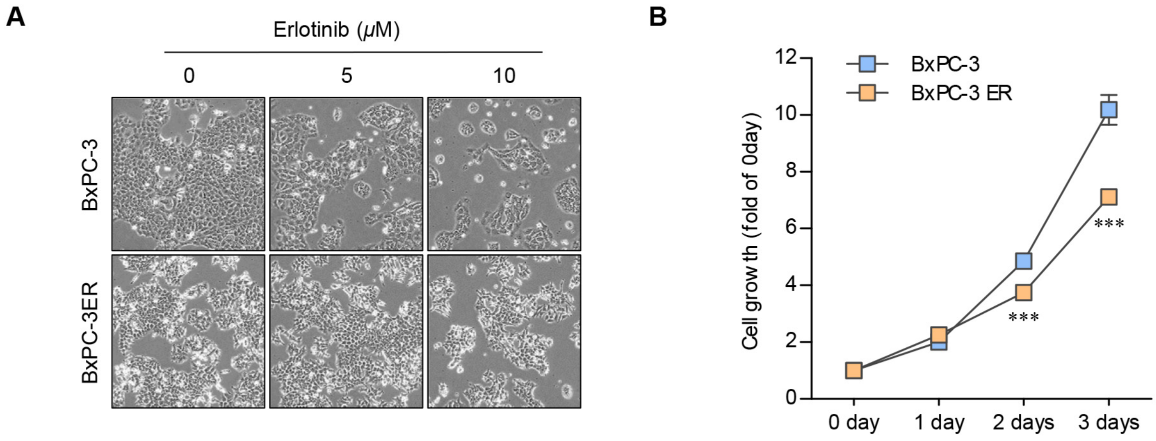

Supplementary Figure 1: Differential characteristics of acquired erlotinib resistance in BxPC-3ER cells. (A) Morphological changes in BxPC-3ER cells after acquisition of erlotinib resistance. BxPC-3 and BxPC-3ER cells were plated onto 6-well plates (1.5×10^5 cells/well) and treated with indicated concentrations of erlotinib for 72 h. Microscopic image of BxPC-3 (top) and BxPC-3ER (bottom) cells at ×100 magnification. **(B)** Cell growth curve of BxPC-3 and BxPC-3ER cells. Cells were seeded onto 96-well plates (1×10^3 cells/well) and incubated for 72 h. Cell viability was determined using an MTT assay. Statistical analysis was conducted using a Student's *t*-test. ***$p < 0.001$, compared to the BxPC-3 group. Error bars show mean ± SD (n = 4).

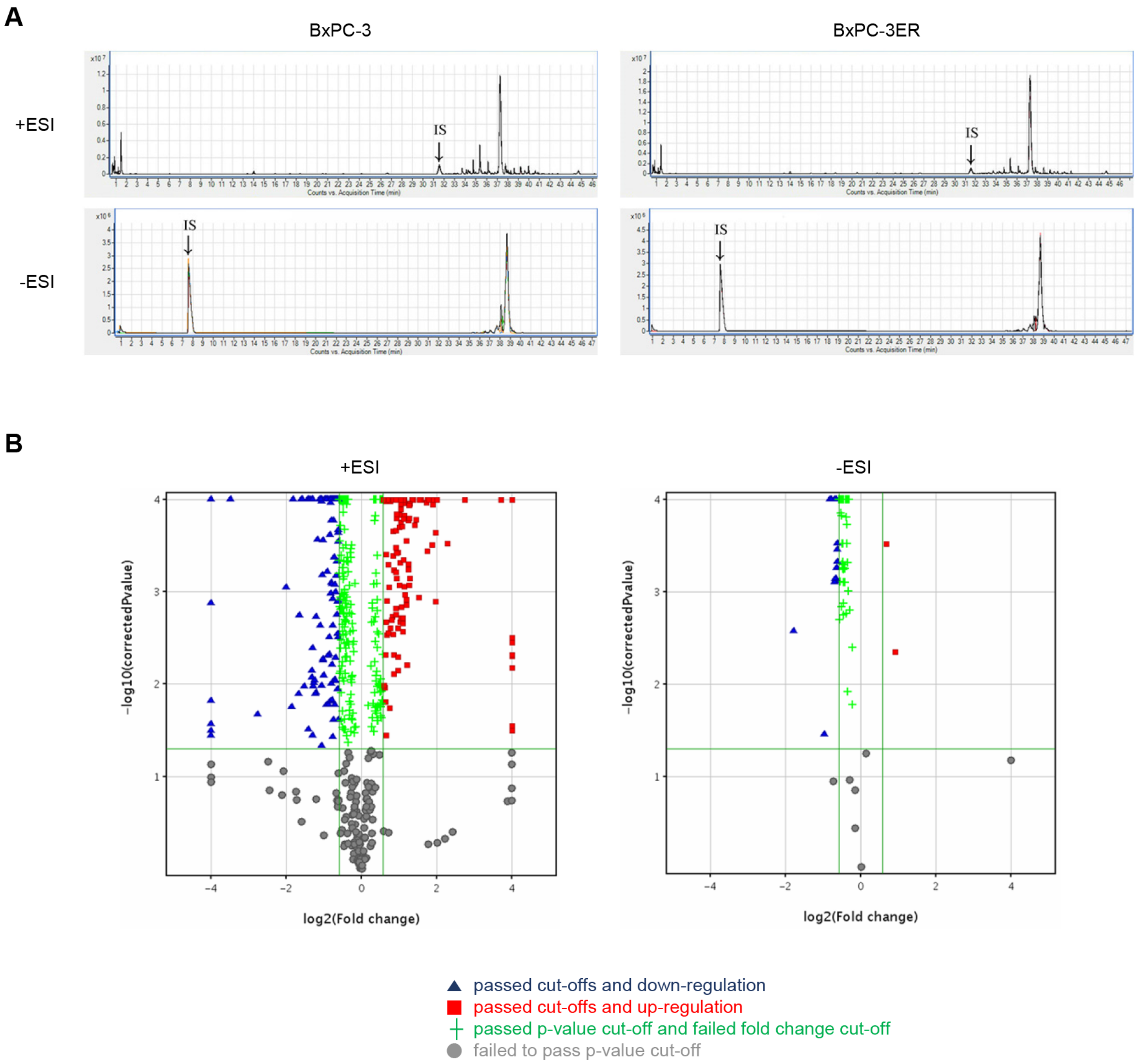

Supplementary Figure 2: Ion features obtained from untargeted metabolomic analysis. **(A)** Overlay of LC-QTOF-MS total compound chromatograms of metabolites in BxPC-3 and BxPC-3ER cells (n = 5 for each). **(B)** Volcano plots of fold changes and *p*-values in metabolite data sets from BxPC-3 and BxPC-3ER cells.

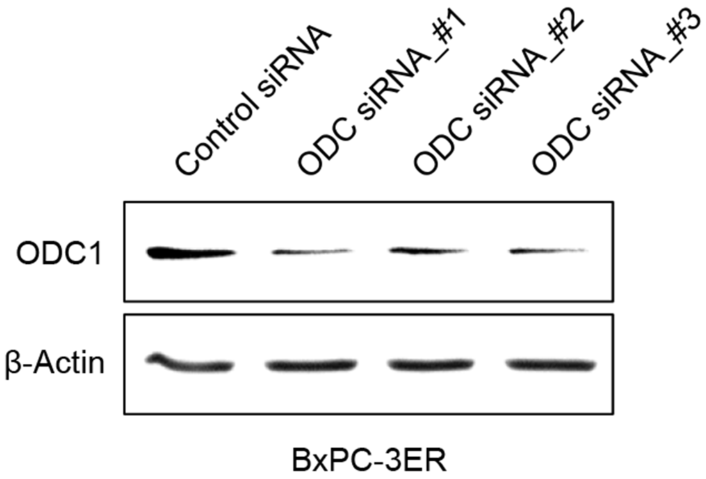

Supplementary Figure 3: Knockdown of ODC1 by small interfering RNAs (siRNAs) in BxPC-3ER cells. BxPC-3ER cells were plated onto 6-well plates (2×10^5 cells/well) and transfected with 100 pmole of 3 different double-stranded siRNAs against ODC1. Total protein was isolated 48 h after transfection and analyzed by western blot.

Supplementary Table 1: Significantly changed metabolites in the BxPC-3 and BxPC-3ER cells confirmed by LC-QTOF-MS -based untargeted metabolomic analysis

m/z	t_R(min)	Metabolites	Mass deviation (ppm)	P value	Log2 fold change	Ionization mode	Identification
104.1073	0.81	Choline	-2.97	1.9e-3	0.8	+ESI	MS/MS library
132.0766	0.90	Creatine	1.17	4.6e-5	1.4	+ESI	MS/MS library
204.1125	1.11	Acetylcarnitine	2.62	4.6e-6	3.7	+ESI	MS/MS library
348.0699	1.23	Adenosine-5'-monophosphate	1.33	3.1e-4	1.9	+ESI	MS/MS library
136.0616	1.37	Adenine	1.26	5.7e-4	1.2	+ESI	Standard
137.0450	2.26	Hypoxanthine	5.79	9.1e-11	16.4	+ESI	MS/MS library
268.1037	2.92	Adenosine	1.24	1.5e-1	1.2	+ESI	Standard
274.2732	34.28	C16 Sphinganine	3.13	1.6e-6	-1.6	+ESI	MSC
318.2985	34.4	Phytosphingosine	5.58	2.2e-6	-3.5	+ESI	MSC
323.0285	1.24	Uridine-5'-monophosphate	0.28	1.7e-3	0.9	-ESI	Standard

tR, retention time; MSC, Molecular Structure Correlator.
P values were calculated using un-paired t-test and analysis of variance with a multiple testing correction (Benjamini-Hochberg False Discovery Rate).
Positive and negative numbers in Log2 fold change indicates increase and decrease in BxPC-3ER cells, compared with BxPC-3 cells, respectively.

Supplementary Table 2: Significantly changed metabolites in the BxPC-3 and BxPC-3ER cells confirmed by MS-based targeted metabolomic analysis

Metabolites	p-value	Log2 fold change	Metabolites	p-value	Log2 fold change
Hexose	4.69E-05	-1.78	PC aa C42:5	2.25E-08	-1.65
Taurine	2.56E-12	4.08	PC aa C42:6	6.38E-09	-1.31
Serotonin	8.04E-14	nd	PC ae C30:0	1.41E-07	-1.27
Kynurenine	9.90E-06	nd	PC ae C32:1	3.13E-08	-1.51
Trans-4-Hydroxyproline	1.36E-07	1.89	PC ae C32:2	1.92E-06	-1.04
Asymmetric DMA	3.27E-09	nd	PC ae C34:0	3.81E-10	-2.35
Glutamate	3.26E-09	1.86	PC ae C34:1	3.51E-10	-2.42
Methioninesulfoxide	5.85E-04	nd	PC ae C34:2	9.11E-10	-2.15
Ornithine	1.49E-05	1.07	PC ae C34:3	1.83E-08	-1.65
Putrescine	2.36E-10	5.59	PC ae C36:0	1.31E-08	-1.57
Spermidine	3.41E-09	1.68	PC ae C36:1	2.63E-10	-2.54
Butyrylcarnitine	8.40E-09	2.42	PC ae C36:2	9.93E-11	-2.71
Propionylcarnitine	5.03E-08	2.65	PC ae C36:3	1.19E-10	-2.61
Acetylcarnitine	2.42E-08	2.69	PC ae C36:4	1.15E-08	-1.74
SM C26:1	6.77E-09	-2.1	PC ae C36:5	3.88E-06	-1.05
SM C26:0	8.94E-07	-1.91	PC ae C38:0	1.02E-06	-1.04
SM C24:1	7.13E-09	-1.87	PC ae C38:1	3.26E-11	-2.87
SM C24:0	7.45E-10	-2.3	PC ae C38:2	1.27E-10	-2.6
SM C18:1	1.20E-07	-1.51	PC ae C38:3	2.07E-09	-2.2
SM C18:0	4.45E-08	-1.74	PC ae C38:4	1.17E-10	-2.57
SM C16:0	8.79E-08	-1.42	PC ae C38:5	6.10E-10	-2.16
SM (OH) C24:1	4.84E-06	-1.92	PC ae C38:6	3.15E-09	-1.81
SM (OH) C22:2	2.02E-08	-1.65	PC ae C40:1	4.32E-11	-2.72
SM (OH) C22:1	2.87E-08	-2.07	PC ae C40:2	1.22E-11	-3.02
SM (OH) C16:1	8.73E-10	-2.15	PC ae C40:3	1.58E-10	-2.51
SM (OH) C14:1	3.77E-06	-1.06	PC ae C40:4	2.92E-10	-2.53
PC aa C36:0	2.30E-08	-1.24	PC ae C40:5	2.24E-10	-2.53
PC aa C36:1	2.92E-08	-1.46	PC ae C40:6	2.87E-10	-2.7
PC aa C36:2	3.68E-10	-2.3	PC ae C42:1	2.73E-10	-2.2
PC aa C38:0	3.48E-10	-2.28	PC ae C42:2	1.28E-11	-3.37
PC aa C38:3	1.09E-07	-1.34	PC ae C42:3	1.06E-09	-2.48
PC aa C40:1	1.23E-07	-1.15	PC ae C42:4	7.86E-10	-2.35
PC aa C40:2	3.23E-09	-2.03	PC ae C44:3	6.29E-10	-2.34
PC aa C40:3	3.64E-08	-1.67	PC ae C44:4	8.01E-10	-1.87
PC aa C40:4	4.23E-07	-1.21	PC ae C44:5	1.57E-09	-2.03
PC aa C40:5	1.09E-06	-1.13	PC ae C44:6	7.62E-09	-1.94
PC aa C40:6	3.57E-07	-1.18	LysoPC a C26:0	2.54E-07	1.56
PC aa C42:2	1.40E-08	-1.65	LysoPC a C26:1	1.67E-05	1.06
PC aa C42:4	6.95E-08	-1.59			

lysoPC a, lysophosphatidylcholine acyl; PC aa, phosphatidylcholine diacyl; PC ae, phosphatidylcholine acyl-alkyl; SM, sphingomyelin.
Positive and negative numbers in Log2 fold change indicates increase and decrease in BxPC-3 ER cells, compared with BxPC-3 cells, respectively. ‘nd’ in log2 fold change means the corresponding metabolite was not detected in either BxPC-3 or BxPC-3 ER cells.
